# Complex rearrangement in acute myeloid leukemia M2 with RUNX1/RUNX1T1 fusion involving chromosomes 8, 17 and 21

**DOI:** 10.1186/s13039-021-00541-6

**Published:** 2021-05-21

**Authors:** Shiba Ranjan Mishra, Leena Rawal, Moneeb A. K. Othman, Atul Thatai, Aditi Sarkar, Vandana Lal, Saurabh Kumar Bhattacharya

**Affiliations:** 1 Department of Clinical Cytogenomics, National Reference Laboratory, Dr. Lal Path Labs Ltd., Block E, Sector 18, Rohini, New Delhi, 110085 India; 2 Institute of Human Genetics, Jena University Hospital, Friedrich Schiller University, Jena, Germany; 3 Molecular Diagnostics, National Reference Laboratory, Dr. Lal Path Labs Ltd., New Delhi, India

**Keywords:** Variant translocation t(8;21), Acute myeloid leukemia, Molecular cytogenetics

## Abstract

**Background:**

The translocation t(8;21)(q22;q22) is one of the most frequent chromosomal abnormalities associated with acute myeloid leukemia (AML) sub type M2. About 3–5 % of cases with additional chromosomal abnormalities, including structural and numerical ones, are reported to include a complex translocation t(8;21;N).

**Case presentation:**

Here we report a chromosome rearrangement observed in a 19 years-old female diagnosed with AML-M2. When subjected to (molecular) cytogenetic analyses a complex three-way translocation involving chromosomes 8, 17 and 21 was detected, forming not a t(8;21;17) as one would expect. Real time-polymerase chain reaction analysis using 6 AML specific markers showed the presence of *RUNX1*/*RUNX1T1* fusion gene transcripts identical to those found in classical translocation t(8;21) coupled with presence of *FLT3*-*ITD* mutation identified by fragment analysis.

**Conclusions:**

The present case highlights importance of complex rearrangements rarely encountered in AML, suggesting that all involved regions harbor critical candidate genes regulating the pathogenesis of AML, leading to novel as well as well-known leukemia associated chromosomal aberrations.

## Background

It is well known that karyotyping and molecular cytogenetics play very important roles in establishing diagnosis and prognosis in acute myeloid leukemia (AML). Even though banding cytogenetics is limited to detect chromosomal rearrangements at a low resolution, it remains the gold standard for basic genetic analyses in AML. Among cytogenetic rearrangements predictive of most favorable outcomes in AML is the translocation t(8;21)(q22;q22), being detectable in approximately 7–8 % of all AML cases. This translocation results in a fusion gene located on derivative chromosome 8, composed of *RUNX1T1* (also known as *ETO*) on 8q22 and *RUNX1* gene (also known as *AML1*) on 21q22 [1]. *RUNX1* gene has also been reported to fuse with different other genes in both *de novo* and therapy related acute leukemia [2]. The 2016 revision of World Health Organization (WHO) classification for hematolymphoid neoplasms underscores that AML associated with translocation t(8;21) constitutes a distinct AML subtype and is associated with favorable prognosis [3]. However, approximately 3–5 % of AML cases with translocation t(8;21) have variant chromosome partners involved [1]. The clinicopathological features of variant t(8;21) is contradictory discussed in literature and some studies have even reported a favorable outcome for patients with variant translocation t(8;21) [4, 5].

In this study, we elucidate a case with novel variant translocation t(8;21) being observed in context of a complex chromosomal rearrangement. To our knowledge, this is the first case reported here for variant three-way t(8;21) involving short arm of chromosome 17. The study suggests that the detailed characterization of *RUNX1T1* via cumulative approaches, like cytogenetics, molecular cytogenetics and real time PCR is necessary to unveil complex chromosome rearrangements.

## Case presentation

**Fig. 1:**
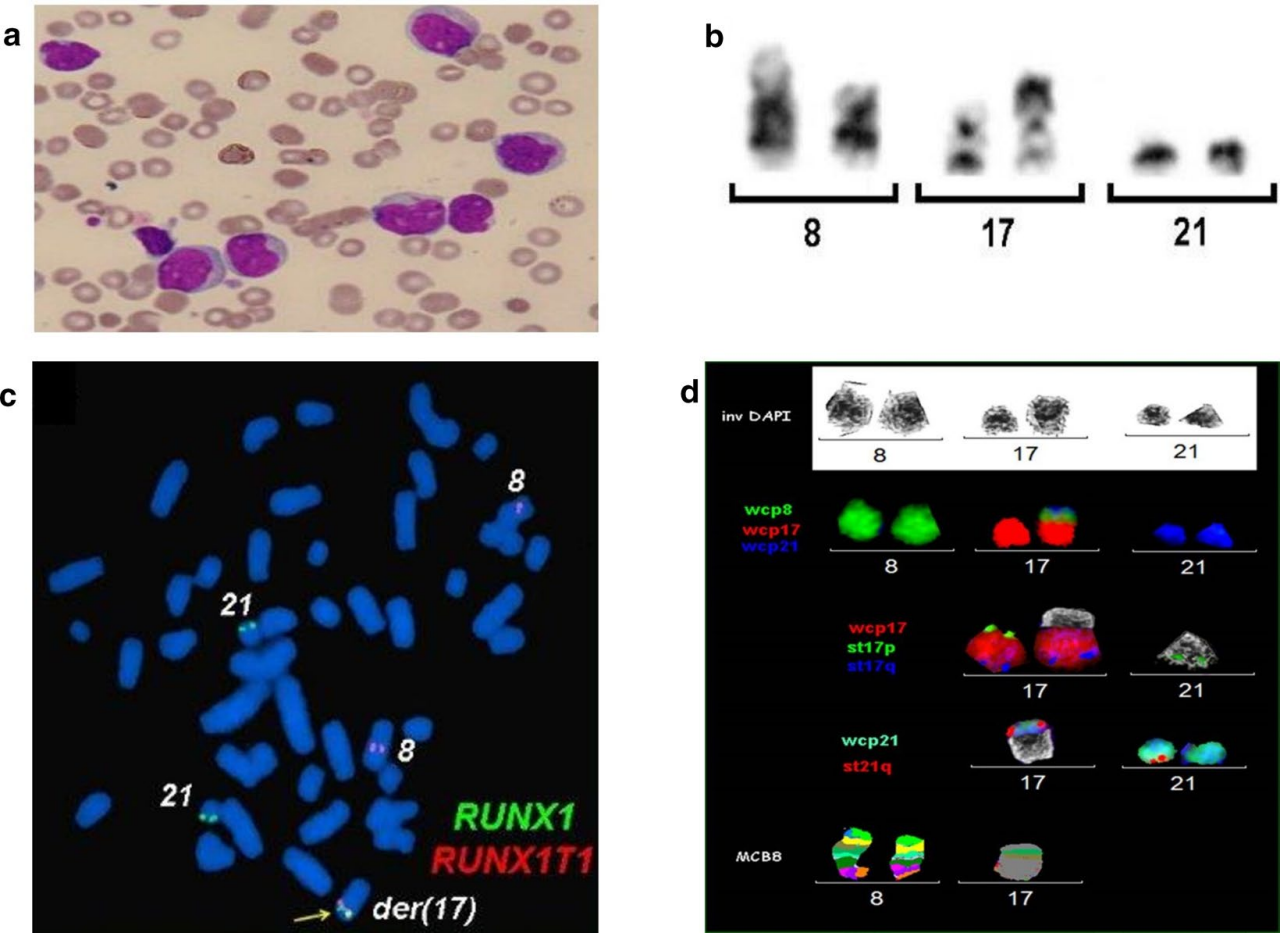
Morphologic and cytogenetic profile. **a** Bone marrow smear aspirate show blast. **b** Partial metaphase spread of G-banded chromosomes. A three way translocation t(8;21;17) at bands 8q22, 21q22 and 17p13 which would be consistent with AML. **c** FISH with the *AML1*/*ETO* dual color, dual-fusion probe. The figures depicts the *RUNX1*/*RUNX1T1* fusion on chromosome 17. **d** FISH with whole-chromosome painting (WCP) for chromosomes 8, 17 and 21. A multi-color banding specific for chromosome 8 and 17, showing the origin and the breakpoints of each rearrangement

In the year 2018, a 19-years-old female of Asian ethnicity belonging to Jammu and Kashmir, India was presented with bleeding gums for the past 8 months and with high fever for 15 days. On physical examination, spleen was palpable with lymphadenopathy in the cervical and inguinal regions. At admission, blood analyses revealed white blood cell (WBC) count of 64.30 × 10^9^/l with 48 % blasts, and platelet count of 95 × 10^9^/l. A bone marrow (BM) examination characterized hypercellular marrow with 65 % leukemic blasts being positive for CD3, CD 15, CD19, CD34, CD-45 and HLA-DR, a clinical statues being compatible diagnosis of AML (Fig. 1a). The distinct morphologic features of marrow blasts, suggested that the patient has CBF-AML. The patient was initially treated with standard induction chemotherapy, including 7 days of infusion with cytarabine (100 mg/m^2^) and 3 days of daunorubicin (60 mg/m^2^). However, complete remission was not achieved after two cycles of chemotherapy. Afterwards she received 12 cycles of low-dose cytarabine and blood transfusions. Following bone marrow biopsy revealed a normal karyotype with ongoing morphologic remission. The patient then received another cycle of cytarabine followed by 2 cycles of 5-azacitidine (75 mg/m^2^ for 7 doses) before discontinuing any further therapy. As the situation stands with over 2 years of diagnosis, the patient remains in complete remission with no evidence of cytogenetic anomalies.

## Methods

### Banding cytogenetics and fluorescence in situ hybridization (FISH)

A BM aspirate collected during initial diagnosis was studied by GTG (Giemsa using trypsin) banding and molecular cytogenetic analyses. Karyotyping was performed at cytogenetics laboratory, LPL (New Delhi, India) according to ISCN 2016 [6]. Fluorescence in situ hybridization (FISH) using the RUNX1/RUNX1T1 probe (dual-color dual-fusion translocation probe) and subtelomeric probes (ST) 17pter, 17qter and 21qter (Vysis/Abbott Molecular, Des Plaines, IL, USA) were also conducted. In-house whole chromosome painting probes (WCP) for chromosomes 8, 17 and 21 and a multicolor banding probe set for chromosome 8 were used to characterize the nature of the complex rearrangement [7].

### DNA extraction

Genomic DNA was extracted from bone marrow samples using QIAsymphony DNA mini kit, Qiagen according to manufacturer’s instruction.

### RNA preparation and cDNA synthesis

Total RNA from the leukemic cells was isolated using QIAamp RNA blood Mini Kit (Qiagen). Total RNA (1–5 µg) was converted to cDNA using High Capacity cDNA reverse transcription kit using random primers as per the established protocols [8].

### Real time detection of AML ETO fusion product

AML-ETO (or RUNX1-RUNX1T1) was detected by Real Time PCR method. The negative and positive controls were analysed in duplicate using ABI 7500 (Applied Biosystems, USA). AML-ETO transcripts were amplified in 20 µl reaction containing 300 nM of each primer, 100 nM of probe and 5 µl of cDNA. PCR master mix was prepared with Premix Ex TaqTM (Probe qPCR) and custom reaction mix containing 0.5 µl of ROX (ROX Reference Dye II; Takara). The PCR program consisted of an initial denaturation at 95 °C for 10 min followed by 40 cycles of denaturation at 95 °C for 15 s, annealing at 60 °C for 1 min and extension. Similarly, endogenous control corresponding to ABL transcripts were amplified in order to compensate for differences in RNA integrity and cDNA synthesis efficiency. The AML-ETO transcripts were amplified using previously published primer probe sequences according to EAC protocol [8]. Standard TaqManTM PCR parameters (ABI 7500) were applied to all AML-ETO and ABL amplifications.

### Fragment analysis

FLT3 gene exon 11 was amplified using the Veriti™ 96 well thermal cycler (Applied Biosystems™) in a 20 µl PCR mixture containing 50 ng of extracted DNA, 0.5 µmol/l each of forward primer with FAM (5′ end-labeled with carboxyfluorescein) labelled and unlabeled reverse primer [8]. The PCR program consisted of an initial denaturation at 95 °C for 10 min followed by 40 cycles of denaturation at 95 °C for 15 s, annealing at 60 °C for 1 min and extension at 72 °C for 10 min. Afterwards, 2 µl of PCR products was mixed with 9 µl HIDI formamide and 0.25 µl ROX500 internal size standard. The mixtures were denatured at 94 °C for 5 min, chilled at 4 °C and then subjected to ABI PRISM 3500Dx Genetic Analyzer. The results were analyzed using the Gene Mapper software (Applied Biosystems).

## Results

GTG banding technique revealed an abnormal putative karyotype of 46,XX,t(8;21;17)(q22;q22;p13) in 20 metaphases analyzed (Fig. 1b). FISH analysis for the *RUNX1*/*RUNX1T1* probe showed 2 orange, 2 green and 1 yellow fusion signals. The latter localized on chromosome 17 indicated a cryptic fusion, *RUNX1*/*RUNX1T1* (co-localization of *ETO* and *AML* signals) on the rearranged chromosome 17 and involvement of third chromosome (Fig. 1c). Whole chromosome paint probes for chromosome 8, 17 and 21 confirmed the presence of a complex chromosomal rearrangement, which was further characterized by FISH-probes shown in Fig. 1d as:

46,XX,del(8)(q21q22),der(17)(21qter->21q22::8q22->8q21::17p13->17qter),der(21)t(17;21)(p13;q22).

**Fig. 2:**
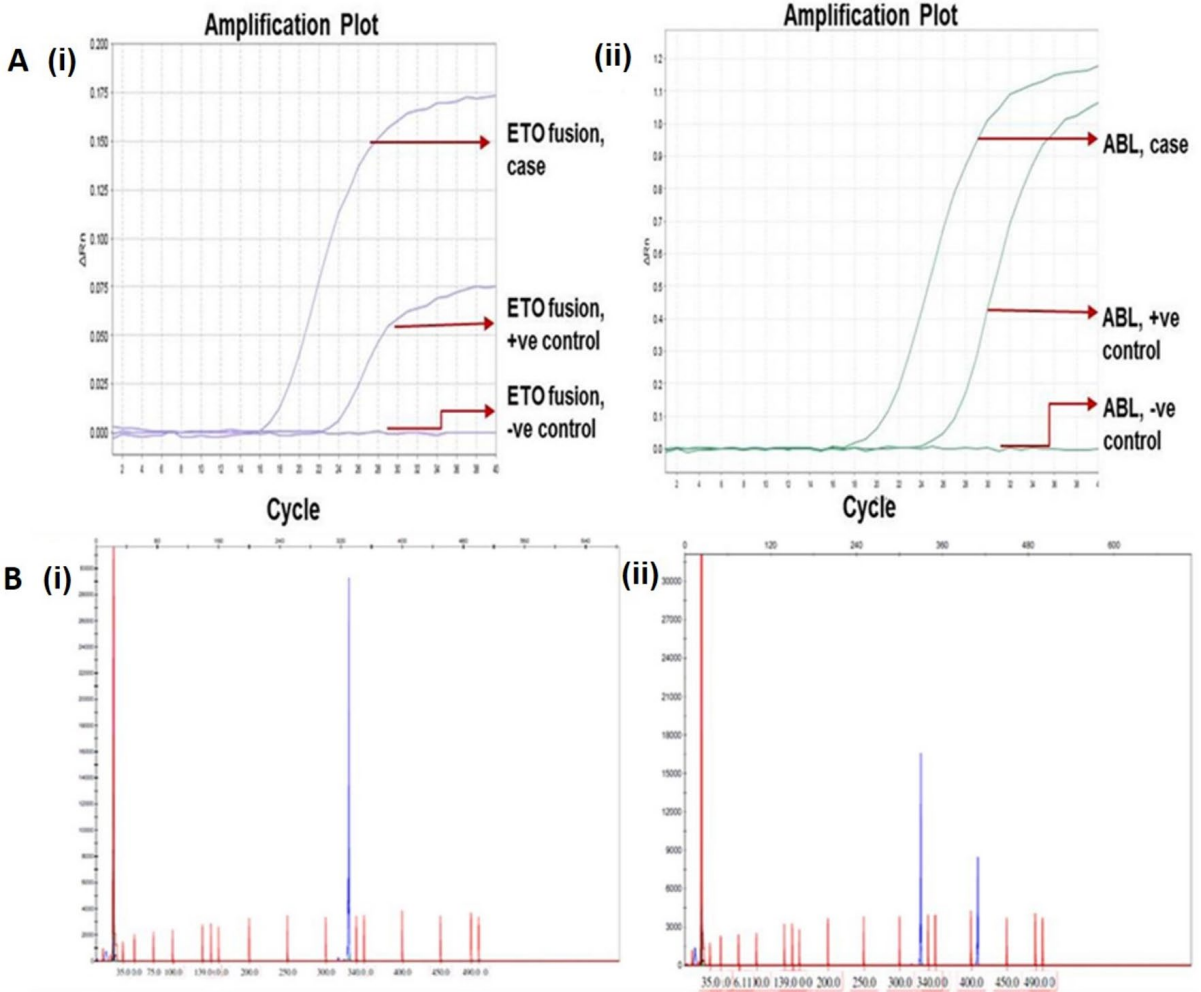
Molecular analysis of leukemia markers. **a** Confirmation of the AML-ETO fusion was done by Taq Man real-time PCR (Amplification plot). (i) The presented case carried AML-ETO fusion translocation t(8:21), thereby confirming the AML/ETO fusion positivity. (ii) The graph for positive and negative controls are shown along with endogenous ABL gene amplification. The x-axis shows the number of PCR cycles and the y-axis shows the normalized fluorescence intensity. The threshold cycle values are calculated automatically by determining the point at which the fluorescence exceeds a fixed threshold line. **b** Fragment analysis method based on capillary electrophoresis for FLT3 INDEL detection. (i) Blue peaks correspond to the FLT3 fragment detection and red peaks correspond to the marker size standards. A wild type patient showing the amplicon size expected. (ii) A mutated patient showed an insertion with an additional peak to the wild type

Further, molecular studies by Real Time PCR confirmed fusion of *RUNX1*/*RUNX1T1* and fragment analysis characterized a *FLT3*-*ITD* mutation in 13q12.2 (Fig. 2a, b).

## Discussion

The translocation t(8;21)(q22;q22) is one of the most common structural chromosomal aberrations in patients with AML, involving fusion of part of the *RUNX1*-gene on chromosome 21 to the almost complete open reading frame of the MTG8/ETO gene on chromosome 8 [2]. The patients with AML have favorable prognosis with achievement of complete morphological remission, and promising respond to high dose Ara-C containing regime [9]. Numerical and structural oddities leading to complex chromosomal anomalies (CCA) in t(8;21) cases involve the variant chromosomes 1, 2, 3, 4, 5, 6, 7, 8, 10, 11, 12, 13, 14, 15, 16, 17, 18, 19, 20, or X [10, 11]. But the prognostic impact of patients with complex variant involvement in addition to t(8;21)(q22;q22) is still unexplored. The CCA in the present study is a novel form of complex translocation involving chromosome 17. We attempt to reinforce a potential relevant role of a der(17) chromosome in the pathogenesis of AML M2, which contains in our patient the *RUNX1/RUNX1T1* fusion gene: karyotype 46,XX,del(8)(q21q22),der(17)(21qter->21q22::8q22->8q21::17p13 > 17qter),der(21)t(17;21)(p13;q22). FISH analysis using TP53 specific probe showed that there is no loss or involvement of TP53 gene in the patient (Additional file 1: Fig. S1).

In addition, Real Time-PCR analysis revealed the presence of *RUNX1/RUNX1T1* fusion transcripts coupled with presence of *FLT3-ITD* mutation. The FLT3 gene mutations are the most frequent genetic abnormality associated with AML. The rapid identification of these mutations is therefore significant to determine the prognostic impact against the overall genetic background of the leukemic cells. The insertion of tandem duplication into exon 11 and exon 12 in the wild-type FLT-3 produces internal tandem duplication (ITD), which results in constitutive activation of the negative regulation of the juxtamembrane domain [12]. Activating the mutation of D835 within the activation loop of FLT-3 in human hematologic malignancies leads to persistent activation of the FLT-3 receptor. In pediatric patients, *FLT3* mutations have been associated with poor prognosis [13]. It has been reported that in adults with CBF (core binding factor) AML the substantial prognostic markers for the outcome are minimal residual disease levels, rather than the *FLT3-ITD* mutations [14].

## Conclusions

The translocation t(8;21)(q22;q22) is a frequent chromosome abnormality seen in AML-M2 which is easily identified by standard chromosome analysis. The present study illustrates a unique complex translocation t(8;21;17), which reinforces that the region on chromosome 17 may harbor a critical candidate gene that could play an important role in the pathogenesis of AML. Additional cases are needed to elucidate its clinical features and prognosis. This finding supports the concept of genetic screening of complex variant translocations in AML patients, thereby envisaging not only the monitoring treatment outcome, but also for clinical manifestation and identification of additional clones that may emerge after the course of treatment.

## Supplementary Information

**Additional file 1: Fig. S1.** FISH analysis using TP53 specific probe: **a** G-banded chromosomes and **b** FISH with TP53 specific probe indicating no loss or involvement of TP53 gene present on chromosome 17 in the patient (shown with arrows).
