# Supplementary figures and images for: Complex rearrangement in acute myeloid leukemia M2 with RUNX1/RUNX1T1 fusion involving chromosomes 8, 17 and 21
Source: Mol Cytogenet. 2021 May 21;14:28. doi: 10.1186/s13039-021-00541-6 (PMC8140419; doi:10.1186/s13039-021-00541-6)

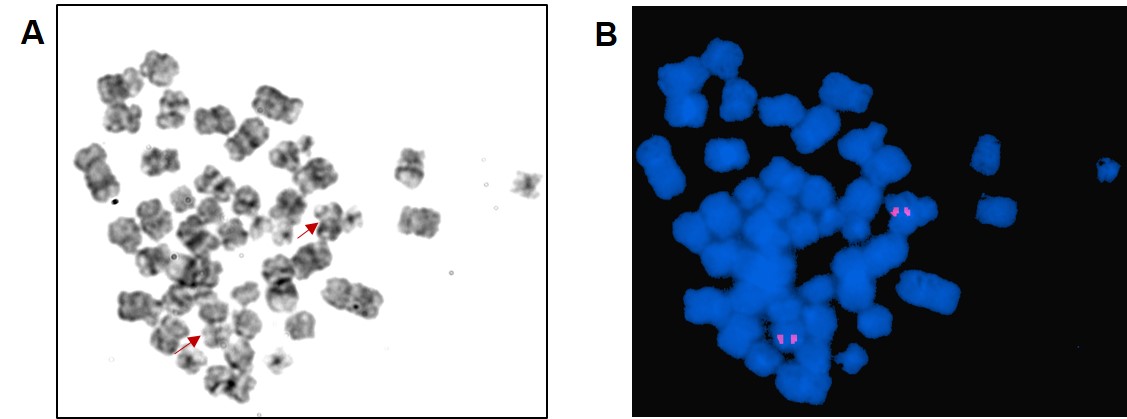

Supplement: Supplementary file 1 — Additional file 1: Fig. S1. FISH analysis using TP53 specific probe: a G-banded chromosomes and b FISH with TP53 specific probe indicating no loss or involvement of TP53 gene present on chromosome 17 in the patient (shown with arrows). [file 13039_2021_541_MOESM1_ESM.jpg]
